# Changes in Follicular CD4+ T Helper Cells as a Marker for Evaluating Disease Progression in the Competition between HIV and Host Immunity

**DOI:** 10.3389/fimmu.2016.00474

**Published:** 2016-10-31

**Authors:** Xiaolei Wang, Widade Ziani, Huanbin Xu

**Affiliations:** ^1^Tulane National Primate Research Center, Tulane University School of Medicine, Covington, LA, USA

**Keywords:** follicular CD4 T helper cells, HIV, TFH cells, SIV, immunity

## Abstract

Follicular CD4+ T helper (TFH) cells interact with B cells in follicular germinal centers and play a prominent role in promoting effective humoral immune responses to pathogens, providing help for B cell development and antibody affinity maturation. Recent studies indicate TFH cells are expanded in HIV/SIV chronic infection, or depleted in terminal stages of disease, yet relatively maintained in elite controllers when compared with uninfected controls. A better understanding of the mechanisms behind these immunologic abnormalities may lead to more effective vaccination and therapeutic strategies. Here, we review recent findings of TFH cells in HIV/SIV infection and discuss the correlation of changes and function of TFH cells with host immunity. Dysregulation or depletion of CD4+ TFH cells likely plays a major role in the inability of HIV-infected patients to mount effective immune responses.

## Introduction

T follicular helper T cells are specialized CD4+ T cells that promote antigen-specific B cell development and maturation. TFH cells represent a heterogeneous cell population including non-GC (germinal center) and GC TFH cells (1–4). Non-GC TFH cells include circulating TFH cells and “immature” PD-1^INT^ TFH cell precursors in lymph nodes, the latter are mostly distributed in interfollicular zones where their differentiation to fully functional GC TFH cells is initiated (5, 6). However, non-GC TFH cells still possess some B-cell helper functions, although they are less efficient than GC TFH cells (7–10). The GC is the crucial niche for the optimal expansion and survival of CD4+ TFH cells and for processes such as somatic hypermutation and selection of high-affinity B cells (11). Mature GC TFH cells (PD-1^HIGH^ CXCR5+CD4+ T cells) are only found in GCs of organized lymphoid tissues such as gut associated lymphoid tissues (GALT), lymph nodes, spleen, and tonsils and are rare or absent in peripheral blood (10). Immature TFH migrate into GCs where they mature and co-localize with follicular dendritic cells (FDC) and B cells (12, 13). Mature GC TFH cells interact with GC B cells by stable cell-to-cell contacts or/and cytokine production such as IL-21 and promote development and maturation of antigen-specific B cells or antibody-secreting plasma cells, thereby ensuring effective, long-term humoral immune responses (14, 15). Mature TFH cells express high levels of CXCR5, PD-1, SLAM-associated protein (SAP), GL7, ICOS, and transcriptional factor Bcl-6 (8, 16). Although peanut agglutinin (PNA) staining is often used to define GC-derived TFH cells in mice and macaques (17), we have found it not specific for TFH in macaque lymph nodes by confocal image analysis. Definitive identification of GC TFH cells in lymphoid tissues is best demonstrated by CXCR5+ PD-1^HIGH^ expression on CD4+ T cells by immunohistochemistry *in situ* (10, 18–20). Interestingly, PD-1 has also been described as a potent T cell inhibitory receptor of CD8+ T cells associated with T-cell “exhaustion” (21, 22); however, its high expression on GC CD4+ TFH cells is involved in the regulation and survival of GC B cells through interaction with its ligands expressed on the latter (13, 23), thus PD-1 is a critical functional molecule for GC TFH cells.

## Architectural Damage of Lymphoid Tissue in HIV Infection

In early HIV/SIV infection, marked lymphoid follicular hyperplasia and dysplasia are observed, and, eventually, massive depletion of CD4 T cells occurs in chronic stages of infection stage. With disease progression, there is generalized lymphoid destruction, as indicated by reduction in GC size and number, loss of the stromal fibroblastic reticular cell (FRC) network, emergence of fibrosis, collagen deposition, and follicular involution (24–27). These features have been shown to gradually result in an inability to mediate antibody production and antigen-specific T cell responses (28–30). Absence of TFH also leads to B-cell apoptosis during priming, thereby preventing B cell differentiation and maturation (31). Thus, loss of CD4+ GC TFH cells in lymphoid tissues is believed to be a major factor in the impairment of B cell responses in HIV infection.

## Infection of GC TFH and Establishment of Persistent Reservoirs in Lymphoid Tissues in HIV/SIV

Organized lymphoid tissues are the major sites for HIV replication and latency (32–34). These and other studies indicate follicular CD4+ T cells in GC in particular may be the major persistent reservoir in patients on ART, which may be directly related to the impairment of effective antibody responses (35). Infected TFH cells residing within these GC “sanctuaries” might be shielded from virus-specific cytotoxic T cell (CTL) responses, allowing them to persist in GC, even when plasma viral loads are completely suppressed by ART (36), p. 1562 (19, 34, 37–42). Further, lower concentrations of antiviral drugs have been demonstrated in lymphoid tissues compared to blood, which may contribute to the persistent viral replication and latent infection in these tissues (43).

Mature GC TFH cells are clearly infected in HIV/SIV (12, 39). We have found that extracellular CCR5 is predominantly expressed on PD-1^INT^ TFH cell precursors, but downregulated on PD-1^HIGH^ GC TFH cells in lymph nodes of uninfected or SIV-infected macaques (12). Since GC TFH cells also do not express other known alternative SIV co-receptors (CXCR6 and GPR15) (39), we have proposed that TFH precursors in the mantle zones or/and T-cell zones might be the major targets for direct viral infection. These immature TFH cell precursors (PD-1^Neg/INT^ CD4+ T cells) in lymph nodes from normal macaques are able to differentiate into mature PD-1^HIGH^ GC TFH cells when stimulated with proinflammatory cytokines, such as IL-6 and IL-21, *in vitro*. When TFH cell precursors, sorted from SIV-infected macaques, differentiate into GC TFH cells stimulated by these cytokines, SIV DNA is detectable in these GC TFH cells, supporting the hypothesis that virus-infected GC TFH cells may develop from migrating TFH precursors that are infected in the non-GC regions, where they express CCR5 (12). Further, our data indicate that SIV RNA/SIV p28 protein levels are relatively lower in GC regions compared with the cortex, paracortex, and medulla of SIV-infected macaques, whereas higher levels of SIV proviral DNA and, to some extent, SIV RNA in GC TFH cells are still detected, suggesting that GC TFH cells are both latently and productively infected by HIV/SIV (12, 34).

It is well known that abundant cytokines and chemokines are induced as proinflammatory responses to viral infection in acute and chronic HIV/SIV infection (44). Persistently high levels of cytokines such as IL-6, IL-21, and IFN-γ in lymph nodes lead to abnormal accumulations of GC TFH cells (5, 45). In addition, persistent antigen presentation may promote GC TFH cell development (4, 46, 47), and even redirect Th1 cells to differentiate into TFH cells during persistent viral infections such as LCMV infection (37). Recent studies indicate the viral reservoir is rapidly established during the “eclipse” phase, prior to SIV viremia (48), and this may be associated with increased cell activation, aberrant TFH cell differentiation, and proinflammatory responses at this stage. Together, these findings suggest TFH cell precursors are infected as they migrate toward GCs, where mature GC TFH cells display productive and latent infection. Thus, persistent HIV/SIV replication and infection and chronic systemic immune activation (49) accompanied with elevated proinflammatory cytokine responses, and persistent antigen stimulation, drives TFH precursor activation, migration to and infection within the T-cell zones, and subsequent migration into the GC where GC TFH fully mature, lose CCR5 expression, and persist as productively and latently infected cellular reservoirs for HIV/SIV infection. Finally, these GC TFH cells may persist in what may be an immune privileged site, which may not be accessible to ART or antiviral cellular responses. Eliminating virus from these “sanctuary” sites may be a significant hurdle for vaccination and cure strategies.

## Changes in TFH Cells Depend on the Consequence of HIV Infection and Host Immunity

Previous studies indicate that TFH cells in lymph nodes are expanded in HIV- or SIV-infected individuals (17, 50), whereas others reported that TFH cell are significantly depleted during HIV infection in peripheral blood and spleen (13, 51–54). By examining large numbers of SIV-infected macaques, our studies show that GC TFH cells are significantly depleted by 14 dpi, and then gradually accumulate in the chronic stage of infection. However, we do not find consistent expansions of GC TFH in all chronically SIV-infected macaques. Further analysis indicate that GC TFH cells accumulate in chronic SIV/HIV infection in adult animals infected with pathogenic SIVmac (asymptomatic chronic), whereas there were little to no changes in these cells in animals inoculated with less pathogenic viruses or those associated with control of viremia (i.e., Mamu-A*01+ rhesus macaques) when compared with uninfected macaques. Of note, adult animals with AIDS (opportunistic infections or neoplasia) show marked losses of GC TFH cells and higher levels of turnover, activation, and apoptosis correlating with chronic inflammation (13). Effective immune responses ultimately resolve viral infections, whereas immune deficiencies fail to clear virus and lead to persistent infections (22, 55). Ours and other studies demonstrate GC TFH cells are enriched in lymph nodes of chronically SIV-infected animals, accompanied by reductions of PD-1^NEG/INT^ CD4+ T cells and increased levels of dysfunctional PD-1+ CD8+ T cells with disease progression (13, 18). In comparison, percentages of PD-1+ CD8+ T cells are lower in uninfected or SHIV-infected cohorts. Finally, untreated infants infected with HIV maintain higher viremia throughout infection and rapidly progress to AIDS (56, 57) which emerging evidence suggests may be due to inadequate TFH responses. Combined, these findings suggest persistent SIV infection leads to exhaustion of TFH precursors and abnormal accumulation or ultimate depletion of GC TFH cells, concomitant with increased dysfunctional CD8+ T cells, leading to eventual disease progression. As compensatory responses, GC TFH cells excessively differentiate, and remain productively and/or latently infected, until they are depleted in terminal AIDS. Together, data suggest that changes in GC TFH cell number and function closely parallel stages in the progression of disease.

## Dysfunction of TFH Cells

**Figure 1:**
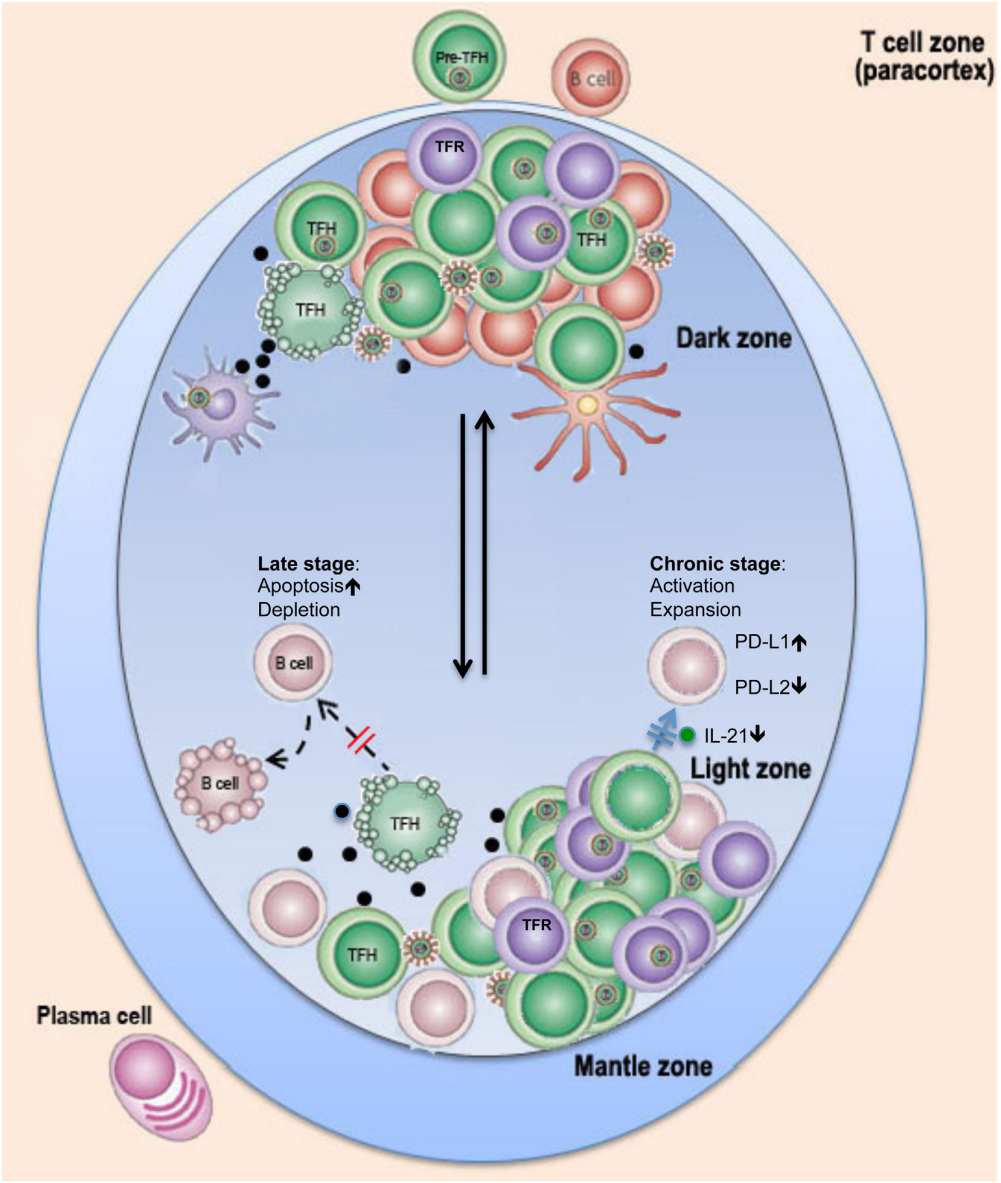
**Schematic of possible changes in TFH cells in lymph nodes in pathogenic HIV infection**. Rapid flux of GC B cells between the dark and light zones facilitates several iterative rounds of mutation and selection, resulting in the generation of memory B cells and plasma cells with high-affinity antibodies. In HIV/SIV infection, follicular dendritic cells (FDC) in lymph nodes are exposed/infected during HIV infection and secrete high levels of proinflammatory cytokines, which, in combination with viral antigens, promote GC TFH and TFR cell expansion in chronic stage or ultimate depletion of GC TFH cells by direct lysis or/and apoptosis at later stages. Loss of help or dysfunction of TFH cells leads to impairment of B cell function and germinal center reaction and prevents long-term effective humoral immune responses to HIV infection.

Constitutive and high expression of PD-1 on GC TFH cells promotes IgG production possibly through PD-1/PD-L2 interactions, suggesting PD-1 regulates B-cell functions in GC *via* direct cell-to-cell interaction (10, 13). Other reports indicate engagement of PD-1 on TFH cells inhibits IL-21 production in HIV infection, resulting in inadequate B-cell help indirectly through the PD-1/PD-L1 pathway (38), which is supported by decreased levels of IL-21 production in TFH cells in chronic SIV infection. Thus, PD-L1 upregulation and PD-L2 downregulation on B cells, which are observed in chronic HIV/SIV infection, might result in impairments of B-cell function and antibody production in chronic HIV/SIV infection (13). B-cell follicles contain a novel subset of regulatory T cell (Treg), termed follicular regulatory T cells (TFR), which express CXCR5 and repress effective GC responses through interactions with TFH cells (58–60). Recent studies reported that TFR cells are expanded and impair TFH functions in HIV/SIV infection (61, 62). As discussed above, factors such as architectural disruption of lymphoid tissues, aberrant TFR regulation, dysregulation of B cells, TFH cell infection, and eventual TFH depletion in AIDS are all fundamental contributors to the impairment of functional B-cell responses and antibody production during HIV/SIV infections (Figure 1). Antiviral therapy, in combination with anti-inflammatory agents and perhaps inhibitors of cell differentiation, could be considered as an adjunct to early intervention strategy to reduce viral reservoir size and lymphoid tissue disruption and improve humoral immune responses in HIV-infected patients.

## Author Contributions

HX wrote and revised the manuscript; XW and WZ assisted with manuscript preparation.

## Conflict of Interest Statement

The authors declare that the research was conducted in the absence of any commercial or financial relationships that could be construed as a potential conflict of interest.
